# Efficacy and safety of rituximab treatment in patients with idiopathic inflammatory myopathies: A systematic review and meta-analysis

**DOI:** 10.3389/fimmu.2022.1051609

**Published:** 2022-12-12

**Authors:** Chao Zhen, Ying Hou, Bing Zhao, Xiaotian Ma, Tingjun Dai, Chuanzhu Yan

**Affiliations:** ^1^ Research Institute of Neuromuscular and Neurodegenerative Diseases and Department of Neurology, Qilu Hospital, Cheeloo College of Medicine, Shandong University, Jinan, China; ^2^ Department of Neurology, Qingdao Municipal Hospital, School of Medicine, Qingdao University, Qingdao, China; ^3^ Department of Neurology, Qilu Hospital (Qingdao), Cheeloo College of Medicine, Shandong University, Qingdao, China; ^4^ Department of Medicine Experimental Center, Qilu Hospital (Qingdao), Cheeloo College of Medicine, Shandong University, Qingdao, China; ^5^ Department of Central Laboratory and Mitochondrial Medicine Laboratory, Qilu Hospital (Qingdao), Cheeloo College of Medicine, Shandong University, Qingdao, China; ^6^ Brain Science Research Institute, Shandong University, Jinan, China

**Keywords:** rituximab, idiopathic inflammatory myopathies, meta-analysis, efficacy, safety

## Abstract

**Objective:**

Idiopathic inflammatory myopathies (IIMs) are a heterogeneous group of autoimmune diseases with various subtypes, myositis-specific antibodies, and affect multiple systems. The treatment of IIMs remains challenging, especially for refractory myositis. In addition to steroids and traditional immunosuppressants, rituximab (RTX), a B cell-depleting monoclonal antibody, is emerging as an alternative treatment for refractory myositis. However, the therapeutic response to RTX remains controversial. This meta-analysis aimed to systematically evaluate the efficacy and safety of RTX in patients with IIMs, excluding sporadic inclusion body myositis.

**Methods:**

PubMed, Embase, Cochrane Library, China National Knowledge Infrastructure, and WanFang Data were searched for relevant studies. The overall effective rate, complete response rate, and partial response rate were calculated to assess the efficacy of RTX. The incidences of adverse events, infection, severe adverse events, severe infection, and infusion reactions were collected to evaluate the safety of RTX. Subgroup analyses were performed using IIM subtypes, affected organs, continents, and countries. We also performed a sensitivity analysis to identify the sources of heterogeneity.

**Results:**

A total of 26 studies were included in the quantitative analysis, which showed that 65% (95% confidence interval [CI]: 54%, 75%) of patients with IIMs responded to RTX, 45% (95% CI: 22%, 70%) of patients achieved a complete response, and 39% (95% CI: 26%, 53%) achieved a partial response. Subgroup analyses indicated that the overall efficacy rates in patients with refractory IIMs, dermatomyositis and polymyositis, as well as anti-synthetase syndrome were 62%, 68%, and 62%, respectively. The overall efficacy rates for muscle, lungs, and skin involvement were 59%, 65%, and 81%, respectively. In addition, studies conducted in Germany and the United States showed that patients with IIMs had an excellent response to RTX, with an effective rate of 90% and 77%, respectively. The incidence of severe adverse events and infections was 8% and 2%, respectively.

**Conclusion:**

RTX may be an effective and relatively safe treatment choice in patients with IIMs, especially for refractory cases. However, further verification *via* randomized controlled trials is warranted.

## 1 Introduction

Idiopathic inflammatory myopathies (IIMs), collectively known as myositis, are a heterogeneous group of acquired autoimmune-mediated myopathies that may be classified into the following subtypes: dermatomyositis (DM), polymyositis (PM), sporadic inclusion body myositis (sIBM), anti-synthetase syndrome (ASS), immune-mediated necrotizing myopathy (IMNM), and overlap myositis (1–3). IIMs are a group of multisystem diseases that may affect multiple organs other than muscles, including the skin, lungs, joints, or even the heart. The annual incidence rate ranges from 11 to 660 per 1,000,000 person-years (1). Treatment mainly involves the use of glucocorticoids, in combination with other immunosuppressive agents. Owing to the wide phenotypic heterogeneity, the therapeutic effect varies. The treatment of IIMs, especially refractory myositis, remains a challenge.

Rituximab (RTX) is a human/chimeric, monoclonal antibody with a specific affinity for CD20, a B-lymphocyte transmembrane protein, and usually leads to the depletion of peripheral B lymphocytes, lasting 6–9 months in patients with lymphoma (4). RTX depletes CD20+ B cells *via* at least four mechanisms: antibody-dependent cell-mediated cytotoxicity, complement-mediated cytotoxicity (CDC), antibody-dependent phagocytosis and direct apoptosis (5). RTX has been approved by the Food and Drug Administration (FDA) for rheumatoid arthritis and antineutrophil cytoplasmic antibody (ANCA)-associated vasculitis (AAV) (6, 7). Moreover, increasing evidence for the efficacy of RTX in various other rheumatic inflammatory diseases has been reported over the past two decades. Based on the European League Against Rheumatism (EULAR) recommendations for the management of SLE, RTX may be considered to treat organ-threatening, refractory SLE manifestations, such as nephritis and neuropsychiatric diseases (8, 9). Two randomized placebo-controlled phase 2 trials demonstrated the benefits of RTX in patients with relapsing-remitting MS (RRMS) and primary progressive MS (PPMS), respectively (10, 11). The efficacy and safety of RTX in MS were also validated in a large multicenter cohort study in Sweden (12).

Increasing evidence suggests that B cells may also be involved in IIM pathogenesis. In juvenile DM (JDM), immature transitional B cells expand significantly and are correlated with the type 1 interferon (IFN) signature, which plays a crucial role in innate and adaptive immunity, and is involved in DM. Thus, B cells may play a role in JDM development (13). Moreover, the expression of the TNF family member B cell activating factor (BAFF) is increased in both the serum and muscle fibers of IIM (14, 15). Therefore, B cell depletion may have a favorable effect on IIM. The Rituximab in Myositis (RIM) trial was a large, randomized placebo-controlled clinical trial conducted in 200 refractory adult patients with PM and adult and juvenile DM. Patients were randomized into the RTX early group or RTX late group. Although there was no difference in the time to achieve the definition of improvement (DOI) between groups (the primary end point), up to 83% of patients achieved DOI by the end of the trial (16). In a Colombian cohort, 62% of patients with refractory myositis achieved remission (17). While in an open-label, phase II trial conducted by Allenbach et al., only 20% of patients with ASS achieved the primary endpoint (an improvement of at least two points in at least two different muscle groups) (18). A number of case series and small open-label trials that have reported the efficacy of RTX for refractory myositis. Considering the large outcome differences in outcomes reported in previous studies, we aimed to resolve the limitations of individual trials and systematically assess the efficacy and safety of RTX in patients with IIM in this meta-analysis.

## 2 Methods

### 2.1 Search strategy

Two independent investigators (CZ and YH) conducted a systematic literature search using PubMed, Embase, the Cochrane Library, China National Knowledge Infrastructure, and WanFangData, from their commencement to June 2021. The following search terms were used: “Myositis” OR “Idiopathic Inflammatory Myopathies” OR “Inflammatory Myopathies” OR “Dermatomyositis” OR “Polymyositis” OR “immune-mediated necrotizing myopathy” AND “Rituximab” OR “Mabthera” OR “anti-CD20” OR “Rituxan” (the complete search strategy is provided in the **Supplemental Information**).

### 2.2 Selection criteria

The eligibility criteria were as follows. 1. IIM was diagnosed according to Bohan and Peter’s criteria (19), the 119th European Neuro Muscular Center criteria (20), or the 2017 EULAR/ACR classification for IIM (3). 2. Patients received RTX therapy in any dosage, with or without combination therapy, and part of a study with a sample size of not less than five. 3. The study evaluated the efficacy and/or safety of RTX for the treatment of IIM, and included patients who received RTX and the number of responders and/or the number of patients who experienced adverse effects, or sufficient raw data to allow the calculation of the aforementioned numbers. 4. The study was published in English or Chinese.

The exclusion criteria were as follows. 1. Reviews, meeting abstracts, case reports, and animal experiments. 2. Trials without extractible data. 3. The study included patients with sIBM.

### 2.3 Data extraction and quality assessment

Two investigators independently extracted the following data from the included studies: authors, year of publication, type of study, country, number of cases, IIM subtype of enrolled patients, age, disease duration, RTX regimen, outcome measurements, outcome evaluation time, and follow-up time. Since most of the included articles were single-arm tests, the methodological index for non-randomized studies (MINORS) criteria were used to evaluate the methodological quality of the included studies. For studies without a control group, the MINORS scale was used and consisted of the following items: clear aims, the inclusion of consecutive patients, prospective collection of data, appropriate endpoints to the aim of the study, unbiased evaluation of endpoints, follow-up period appropriate to the major endpoint, loss to follow-up of <5%, and prospective calculation of the sample size, was used. For studies with a control group, the following items were considered: a control group with standard intervention, contemporary groups, comparable baseline equivalence of groups, and statistical analysis adapted to the study design (21), were considered. Each item was scored as 0 (not reported), 1 (reported but inadequate), or 2 (reported and adequate). The optimal total score was 16 or 24.

### 2.4 Statistical analysis

STATA 16.0 was used for the single-group rate meta-analysis. The original data were normalized using the double arcsine method, and the final results were restored using the formula P=(sin(tpda/2))^2^. We used the I^2^ test to evaluate statistical heterogeneity among studies. We used the fixed effects model if I^2^ was <50%, and the random effects model if I^2^ was >50%. We conducted a series of pooled analyses of eligible studies to assess the effective rate of RTX (overall effective rate, complete response rate, and partial response rate) and the safety of RTX (incidence rate of adverse events, infection, severe adverse events, severe infection, and infusion reaction). Subgroup analyses were performed according to the IIM subtypes (ASS, refractory IIM, DM, and PM), affected organs (muscle, lung, and skin), continent (Europe and America), and country (USA, France, UK, and Germany). We also conducted a sensitivity analysis to identify the sources of heterogeneity. A graphical examination of funnel plots and Begg’s test were performed to assess publication bias. A two-tailed *p*-value <0.10 was considered statistically significant in the assessment of heterogeneity.

## 3 Results

### 3.1 Study selection and characteristics of the eligible studies

**Figure 1:**
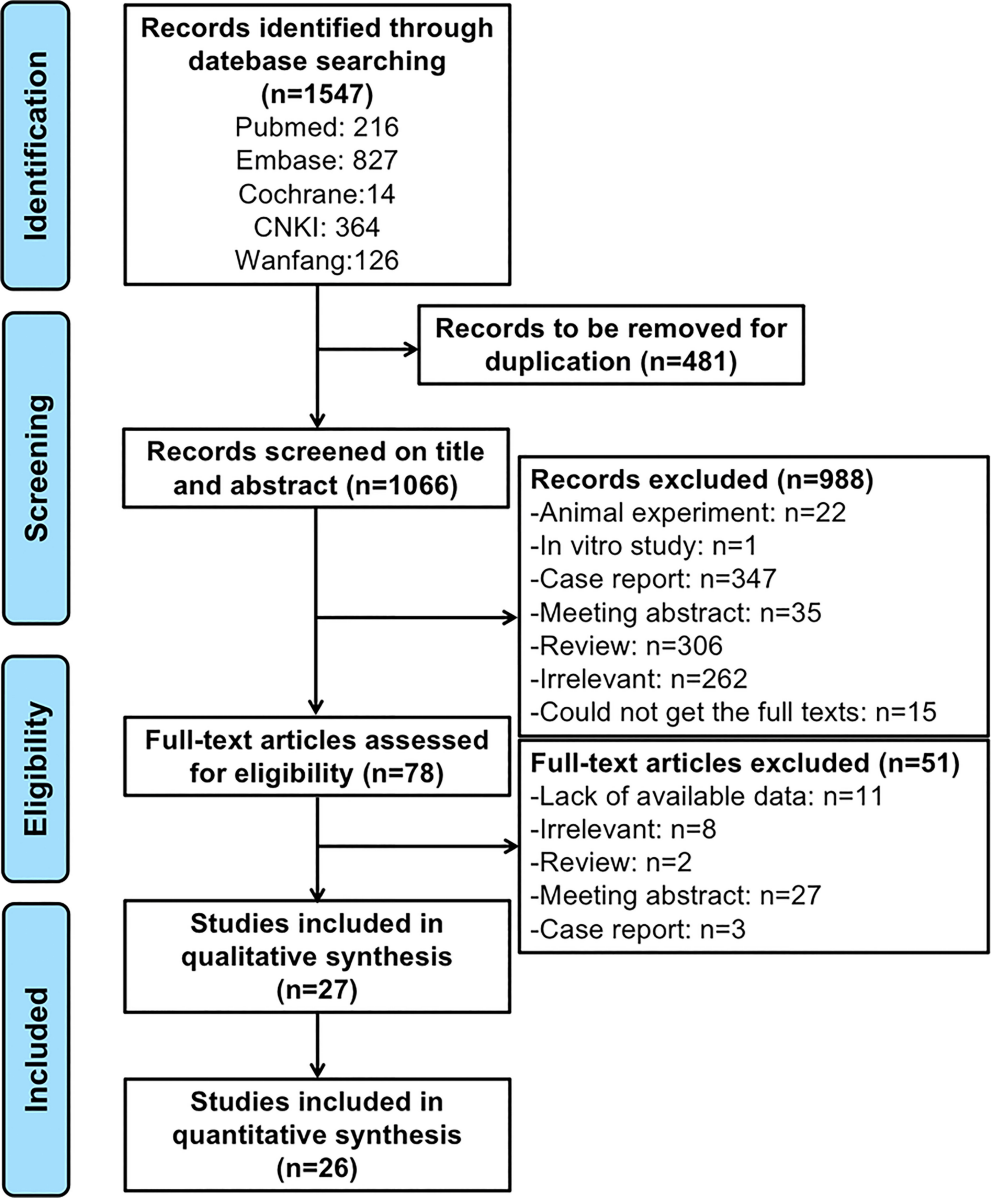
Flow diagram of the study identification and selection process.

A flow diagram of the search process is shown in **Figure 1**. A total of 1547 potentially related articles were identified using the search strategy (216 articles from PubMed, 827 from Embase, 14 from the Cochrane Library, 364 from the China National Knowledge Infrastructure, and 126 from Wanfang). After excluding 481 duplicates, the titles and abstracts of the remaining 1066 articles were screened, and 78 articles were reviewed for full-text screening. Subsequently, 27 articles were included in the qualitative analysis, and one article was excluded because of a low MINORS score (less than 10 points). Ultimately, 26 articles were deemed eligible and were included in the quantitative analysis (16–18, 22–44). Detailed characteristics and the MINORS quality assessments of the eligible studies are presented in **Table 1**. Of the included studies, 18 were conducted in patients with refractory IIM (16–18, 22–25, 28–30, 35–37, 39, 40, 42–44), and 8 included patients with ASS (17, 23, 28, 30, 39, 40, 43). Among the 23 single-arm trials, the quality score of two articles were 14 points, 13 articles were 12 points, one article was 11 points, and seven articles were 10 points.

**Table 1:** Baseline characteristics of included studies.

Author, Year	Type of study	Region	Enrolled patients	Number	Age(years)(mean ± SD)	Disease Duration	RTX regime	Outcome measurements	Evaluation time	Follow-up	MINORS score
Shahin et al, 2021	single center, prospective	Egypt	14DM 8PM	14DM 8PM	Total: 34.9 ± 14.9 (16.0–62.0) years CYC: 35.7 ± 14.8 (16.0–62.0) years RTX: 32.2 ± 16.6 (17.0–59.0) years	NA	4 patients received 3 doses, 1 g each with 6 months apart, 1 patient received only one dose	MRC-SS CK	18 months (after end of RTX)	Total: 7.7 ± 4.3 (0.7–19.0) years CYC:8.5 ± 4.4(0.7– 19.0) years RTX:5.0 ± 2.7(1.0–8.0) years	20
Ahn et al, 2020	retrospective multicenter study	Korea	refractory IIM, 10DM 6PM	Total 16	51.8 (42.5–59.0)years	88.4 (24.3–162.8) months	6 patients received 2 doses of 1g RTX 2 weeks apart, 4 received 2 doses of 0.5 g RTX 2 weeks apart, 3 received a single dose of 1g, 1patient received 0.5g weekly for a total of 4 doses, 1 patient received 0.6g RTX 2 weeks apart, and 1 received a single dose of 0.2g RTX	CPK level daily dose of corticosteroid, physician’s global assessment(PRA).CR PR	12 and 24 weeks after RTX treatment	median 24 weeks, range 24–68 weeks	12
Aggarwal et al, 2016	prospective, randomized, double-blind trial	U.S.A	refractory myositis	72 adult DM, 48 JDM	36.1 ± 19.7 years	5.3 ± 6.9 years	rituximab early (drug at weeks 0/1, placebo at weeks 8/9) or rituximab late arms (placebo at weeks 0/1, drug at weeks 8/9), Rituximab dosing was based on the patient’s body surface area; children with a body surface area 41.5 m^2^ received 575 mg/m^2^ at each infusion, and adults and children with a body surface area >1.5 m^2^ received 750 mg/m2 up to 1 g/infusion	10 cm visual analog scale (VAS)	week 36	36weeks	12
Oddis et al, 2013	Randomized,double-blind, Placebo-Phase Trial	U.S.A	refractory myositis PM,DM,JDM	Total 195	NA	NA	Patients in the rituximab early arm received the drug at weeks 0 and 1, and placebo infusions were given at weeks 8 and 9. Patients in the rituximab late arm received placebo infusions at weeks 0 and 1,and rituximab was given at weeks 8 and 9	improvement prednisone dosage AEs and SAEs	week 44	44weeks	12
Levine et al, 2005	Open-Label uncontrolled Pilot Study	U.S.A	adult DM	6	21–64 years	0.3-15 years	4 intravenous infusions of rituximab given at weekly intervals on days 1, 8, 15, and 22. 3 patients received rituximab at a dose of 100 mg/m^2^/infusion. 3 patients received rituximab at a dose of 375 mg/m^2^/infusion.	muscle strength CPK level FVC Safety	weeks 4, 12, 24,36,and 52	52weeks	10
Ramos-Casals et al, 2010	multicentre study	Spain	refractory IIM : DM PM ASS	Total 20 DM 11 PM 4 ASS 5	49.20 ± 2.98 years (23-77) years	NA	18 patients 375 mg/m^2^/week (x4) 2 patients 1g/15 days (x2)	Overall response Overall response by disease Organ-specific response Adverse events Relapses Deaths	NA	19.00 ± 2.65 (1-52)months	12
Sultan et al, 2008	open-label study	UK	refractory Adult IIM, PM DM	Total 8 2PM 5DM 1JDM	31-63years	14.9 years (range 4-40 years)	1g intravenous infusions on day 0 and day 14.	MMT CPK level	6 months	6 months	12
Bader-Meunier et al, 2011	multicenter prospective cohort study	France	Severe JDM	9	6.2-16 years	3.4 years (range 1 month to 8.4 yrs)	5 patients 4 × 375 mg/m^2^ 1 patient 2 × 375 mg/m^2^ 3 patients 2 × 500 mg/m^2^	MMT CPK level complete clinical response severe infection	NA	1.3 to 3 years	10
de Souza et al, 2018	retrospective single-center cohort study	Brazil	refractory IIM : ASS DM PM	Total 38 13ASS 15DM 10PM	42.6 ± 10.9 years	3.0 (2.0–6.5) years	two infusions (1 g each, 2 weeks apart) and this same scheme was repeated 6 months after the first dose for patients showing no response or stable disease	clinical and laboratory improvements MMT-8, physician’ and patient’ VAS, HAQ and serum levels of muscle enzymes	6 months and 12 months	one-year	12
Chung et al, 2007	open-label, single-arm trial	U.S.A	refractory DM	8	38-76 years	3.5 years (range, 1-24 years)	2 doses of 1 g of rituximab 2 weeks apart	partial remission Muscle Strength:MMT Muscle Enzymes : CPK Skin Disease : DSSI B-Cell Levels Safety	24 weeks	48 weeks	10
Korsten et al, 2020	retrospective observational study	Italy	ASS with ILD	Total 12 RTX 7	NA	NA	2 doses of 1 g of rituximab, 1patient received a 2nd cycle	the alteration of lung parenchyma on HRCT as well as PFTs	NA	31 (6–156) months	18
Sem et al, 2009	a retrospective case series	Norway	ASS patients with severe ILD	11	52 years	1.5-156 months	8 patients two infusions of 1000 mg rituximab, at Days 0 and 14,1 patient received two doses of 700 mg. 2 patients received four weekly infusions of 375 mg/m^2^ body surface.	Pulmonary function tests (PFTs) Safety and adverse effects	6month	≥6 months	12
Mahler et al, 2011	single center, prospective	Netherlands	refractory DM or PM	13	44.4 ± 12.1years	4.0 years (IQR 2.5-6.5 years)	1000 mg i.v., twice, with a 2-week interval, the median number of rituximab courses was 2.0 (IQR 1.5 - 3.5).	CPK LDH levels MMT general health, disease activity and pain, CS dose, functional ability, health-related quality of life and safety	24month	27 months	12
Couderc et al, 2011	multicenter, prospective	France	refractory IIMs	Total 30 12PM 6DM 12ASS	52.5 ± 14.7years (26 - 76 years)	6.1 ± 4.3 years (range 1-18 years)	5 patients 4 × 375 mg 25 patients 2 × 1000 mg	CPK level daily dose of CSs and the opinion of the physician Safety of RTX adverse event infection	NA	17.2 ± 11.3 months (range 1-50 months)	12
Unger et al, 2014	retrospective	Germany	severe, refractory DM or PM	Total 18 13 PM 5 DM	57 ± 18 years	5.4 years (range 0.1- 15)	13 patients 2×1000 mg RTX infusions. 1 patient 4 × 375 mg/m^2^ + 600mg. 2 Patients were switched from 4 × 375 mg/m^2^ to 2×1 g after the first cycle. 1 patient received a single 600 mg RTX infusion.1 patient was treated with a normal 2 × 1000 mg course first and with a reduced dose of 1 × 1000 mg in further courses.	glucocorticoid dose, creatine phosphokinase (CPK) and lung function tests, serious adverse events	50week	2.5 ± 1.6 years (range 0.5-5.4)	10
Marie et al, 2012	retrospective	France	ASS-associated interstitial lung disease	7	57 years (47-59 years)	12 months (8-60 months)	2 infusions of 1 g at days 0 and 14; and a third infusion of 1 g at 6-month follow-up.	PFT CK safety prednisone dose	12 month	12 months	12
Meyer et al, 2015	retrospective case–control study	France	ASS	8	50.4 ± 7.4 years	NA	NA	joint lung musle improvement	NA	93.19 ± 61.31months	11
Leclair et al, 2018	prospective	Canada	IIM	Total 43 27 ARS-ab positive IIM 16 ARS-ab negative IIM	ARS-ab+ 57 ± 10 years ARS-ab- 57 ± 19 years	(months) median (IQR) ARS-ab+ 15 (4-52) ARS-ab-69 (9-166)	500 mg to 1000 mg given 2 weeks apart or 750 mg/m^2^ weekly for two doses or 375 mg/m^2^ weekly for four doses (induction); or any following administration separated from the previous cycle by more than three months (maintenance)	MMT-8 HAQ VAS EQ-5D ACR/EULAR improvement glucocorticoid doses respectively adverse events	at 5-10 months after the first and last RTX cycles	5-10 months	20
Allenbachet al, 2015	open-label, prospective, multicenter	France	Refractory ASS	10	51 years (range, 18–57 years)	0.5-8years	patients received two 1 g infusions of rituximab separated by 2 weeks, followed by 1g infusion 6 months after the day 15 injection was performed.	muscle strength improvement in ILD Adverse events	12month	12months	10
Santos et al, 2021	retrospective	Colombia	Refractory myositis	Total 18 15DM 2PM 1JDM	40.5 years (IQR, 31–49 years)	21 months (IQR, 6.5–40.5 months)	1 g at day 0 and day 15 5 patients 1 circle, 8 patients 2 circles, 2 patients 5 circles, 2 patients 9 circles, 1 patient 11 circles	Clinical, Serological	NA	11 months (IQR, 4–57 months)	12
Muñoz-Beamud et al, 2013	retrospective	UK	DM and PM	16	51.1 years (range 30–62 yrs)	9.75 years (range 2–44 yrs)	2 doses of 1 gram RTX infusions two weeks apart.	Clinical outcomes were measured using the MITAX	6month	NA	12
Bauhammeret al, 2016	retrospective cohort study	Germany	Jo1 Antibody–associated ASS	the RTX group 18	median (range) RTX group 50.9 (26–78)	NA	patients received on average 4.6 cycles of RTX (range 1–13) in a mean interval of 6.4 months	Clinical, CK, Prednisolone-equivalent doses	NA	35 months	10
Landon-Cardinal et al, 2018	retrospective case series	France	anti-HMGCR IMNM	9	43 years	0.75-23years	most patients were administered 2 doses of 1 gram RTX infusions two weeks apart. One patient received a dose of 375 mg/m^2^/body surface area once weekly for 4 weeks. Patients were subsequently re-perfused with RTX 1 g every 6 months at the discretion of the treating physician.	muscle strength CK level	NA	NA	12
Ge et al, 2020	retrospective	China	anti-MDA5 DM	11	39 years (24–59 years)	NA	Seven patients received intravenous RTX (375 mg/m^2^) at 0 and 14 days. Three patients were administered with intravenous RTX (100 mg) at 0, 7, 14, and 21 days. One patient was treated with RTX (100 mg) at 0 and 7 days	Skin ILD infection	NA	≥12 months	14
Andersson et al, 2015	retrospective	Norway	ASS-associated severe interstitial lung disease	24	28-78 years	11-440months	The mean number of Rtx cycles was 2.7 (range 1-11). The first cycle of Rtx treatment was given as one infusion of 1000 mg on each of days 0 and 14, except for three patients (#1, #7 and #11). Patients #7 and #11 were treated according to standard lymphoma protocol (4 × 375 mg/m^2^), while patient #1 was treated with a reduced dose because of perceived infection risk.	ILD MMT8 CK Serious adverse events and mortality	NA	median 52 months	10
Behrens Pinto et al, 2020	prospective	Brazil	ASS	16	43.1 ± 10.1 years	1.5 (0.0–5.8) years	Rituximab treatment consisted of two infusions (1 g each, 2 weeks apart), and this same scheme was repeated every 6 months for 2 years in patients showing clinical response.	MMT-8 HAQ VAS glucocorticoid doses lung computed tomography pulmonary function testing	6 and 12 months	12 months	14

DM, dermatomyositis; PM, polymyositis; CYC, cyclophosphamide; RTX, rituximab; NA, not available; MRC-SS, Medical Research Council sum score; CK, creatine kinase; IIM,idiopathic inflammatory myopathy; CPK, creatine phosphokinase; CR, complete response; PR, partial response; JDM, juvenile dermatomyositis; AE, adverse event; SAE, severe adverse event; FVC, forced vital capacity; ASS, antisynthetase syndrome; MMT, manual muscle testing; VAS, visual analogue scale; HAQ, health assessment questionnaire; DSSI, dermatomyositis skin severity index; ILD, interstitial lung disease; HRCT, high-resolution chest computed tomography; PFT, pulmonary function tests; LDH, lactate dehydrogenase; CS, corticosteroids; ARS-ab, anti-tRNA synthetase autoantibodies; ED-5Q, EuroQol five Dimensions; IQR, interquartile range; HMGCR, Anti-3-hydroxy-3-methylglutaryl-coenzyme A reductase; IMNM, immune-mediated necrotizing myopathy; MDA-5, melanoma differentiation-associated gene 5.

### 3.2 Efficacy of RTX treatment

#### 3.2.1 Complete response rate

**Figure 2:**
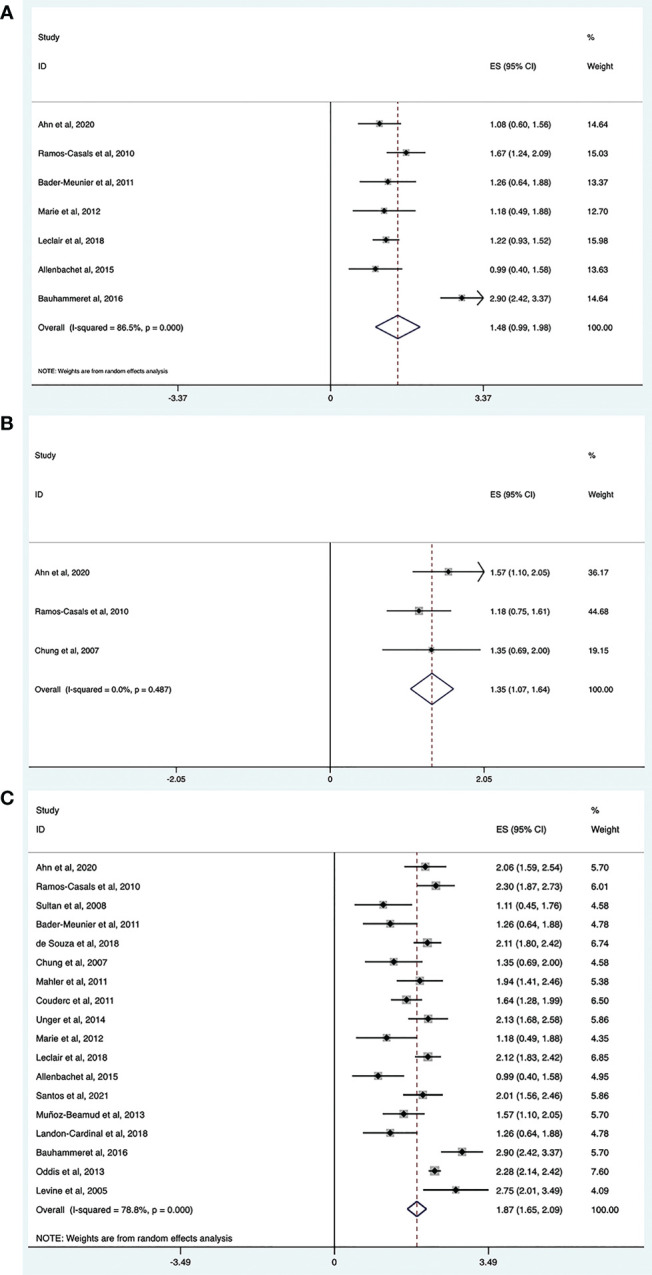
Meta-analysis results of efficacy for RTX in IIMs. Complete response rate **(A)**, partial response rate **(B)** and overall effective rate **(C)**.

The complete response rate was determined from seven trials (n = 121), and the pooled estimate of effectiveness was 45% (95% CI: 23%, 70%) (**Figure 2A** and **Table 2**). Significant heterogeneity was found (*I^2^* = 86.5%, *P* = 0.000). After we conducted the sensitivity analysis, one study was omitted because the study population included patients with Jo-1-associated ASS and the patients received an average of 4.6 cycles of RTX, which may lead to a high response rate (22). Following the omission, the heterogeneity was resolved (*I^2^* = 0, *P* = 0.425) and the complete response rate became 35% (95% CI: 26%, 44%). Notably, although most included studies defined complete response and partial response based on clinical status, the daily dose of corticosteroid, CK level, and/or physician opinion, the details of the criteria differed. Since most of included studies only provided the numbers of patients who achieved complete or partial responses, and specific values of the above index for every patient were not available, we could not set a unified standard to recalculate the numbers. Therefore, for this meta-analysis, we could only directly extract the number of complete and partial responses from the original studies, which may contribute to heterogeneity.

**Table 2:** A pooled results summary of response rate, safety analysis, and subgroup analyses for RTX in IIMs.

	Outcome		Number of included references	Number of cases	Overall effects	Transformed overall effects	I^2^ (%)	p value
Response rates	overall effective rate		18	480	1.87 (1.65, 2.09)	0.65 (0.54,0.75)	78.8	0.000
	complete response rate		7	121	1.48 (0.99, 1.98)	0.45 (0.23, 0.70)	86.5	0.000
	partial response rate		3	44	1.35 (1.07, 1.64)	0.39 (0.26, 0.53)	0	0.487
Safety	Incidence of adverse events		7	135	0.88 (0.55, 1.22)	0.18(0.07, 0.33)	74.1	0.001
	Incidence of infection		15	386	1.07 (0.86, 1.27)	0.26 (0.17, 0.35)	52.1	0.022
	Incidence of severe adverse events		7	248	0.57 (0.28, 0.85)	0.08 (0.02, 0.17)	74.8	0.001
	Incidence of severe infection		5	81	0.29 (0.08, 0.5)	0.02 (0.00, 0.06)	0	0.578
	Incidence of infusion reaction		7	245	0.71 (0.48, 0.93)	0.12 (0.06, 0.20)	25.4	0.244
Subgroup analysis (overall response rate)	patients	refractory IIMs	15	412	1.81 (1.58, 2.05)	0.62 (0.50, 0.73)	77.2	0.000
		DM and PM	7	284	1.93 (1.65, 2.21)	0.68 (0.54, 0.80)	69.6	0.003
		ASS	8	80	1.81 (1.39, 2.23)	0.62 (0.41, 0.81)	74.3	0.000
	affected organs	muscle	6	61	1.75 (1.05, 2.46)	0.59 (0.25, 0.89)	88	0.000
		lung-ILD	9	88	1.87 (1.54, 2.21)	0.65 (0.48, 0.80)	61.8	0.007
		skin	3	22	2.23 (1.83, 2.63)	0.81 (0.63, 0.94)	3.8	0.354
	continent	Europe	11	156	1.69 (1.35, 2.04)	0.56 (0.39, 0.73)	80	0.000
		America	6	308	2.13 (1.91, 2.34)	0.77 (0.67, 0.85)	80	0.000
	country	U.S.A	3	209	2.13 (1.49, 2.76)	0.77 (0.46, 0.96)	78.4	0.01
		France	5	65	1.36 (1.12, 1.61)	0.40 (0.28, 0.52)	6.4	0.37
		UK	2	24	1.39 (0.95, 1.84)	0.41 (0.21, 0.63)	21.2	0.26
		Germany	2	34	2.51 (1.75, 3.26)	0.90 (0.59, 1.00)	81.2	0.021

ASS, antisynthetase syndrome; ILD, interstitial lung disease.

#### 3.2.2 Partial response rate

The partial response rate was calculated from three trials (n = 44), and the pooled estimate of effectiveness was 39% (95% CI: 26%, 53%) (**Figure 2B** and **Table 2**). No heterogeneity was observed (*I^2^* = 0, *P* = 0.487).

#### 3.2.3 Overall effective rate

The overall efficacy rate was determined from 18 trials (n = 480). The pooled effectiveness estimate was 65% (95% CI: 54%, 75%) (**Figure 2C** and **Table 2**). The calculation method for the total effective number is as follows. If the studies provided both the number of complete responders and partial responders, the total effective number was taken as the sum of the two. For studies that only provided the number of improved cases, without a specific classification of whether they were complete or partial responders, the total effective number was taken as the former. There was high heterogeneity (*I^2^* = 78.8%, *P* = 0.000), therefore, we conducted a sensitivity analysis, but studies leading to heterogeneity were not found.

### 3.3 Safety of RTX treatment

#### 3.3.1 Incidence of adverse events and severe adverse events

**Figure 3:**
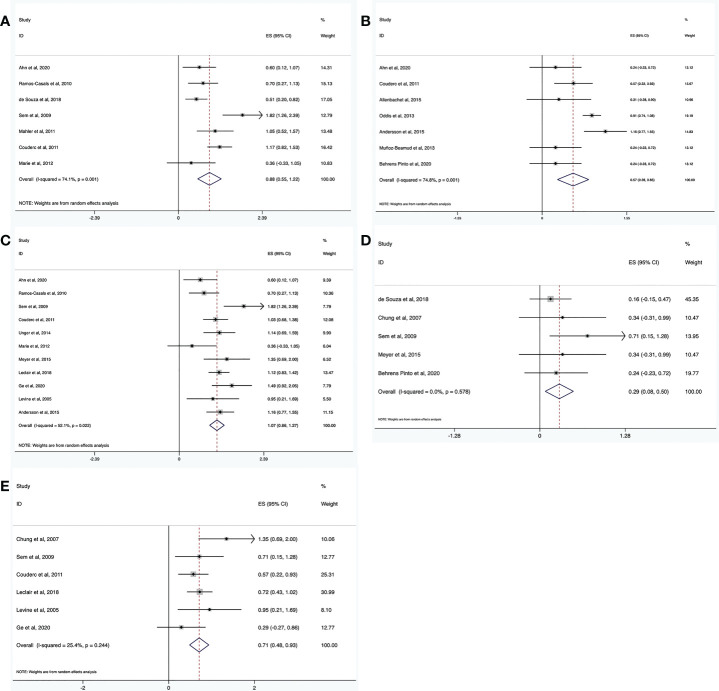
Meta-analysis results of safety for RTX in IIMs. The incidence of adverse events **(A)**, severe adverse events **(B)**, infection **(C)**, severe infection **(D)**, and infusion reaction **(E)**.

Seven trials reported adverse events (n = 135). The pooled incidence estimate was 18% (95% CI: 7%, 33%) (**Figure 3A**, and **Table 2**). Significant heterogeneity was detected (*I^2^* = 74.1%, *P* = 0.001). After the sensitivity analysis, two studies were excluded (30, 41). Consequently, the heterogeneity was resolved (*I^2^* = 0, *P* = 0.45) and the incidence of adverse events decreased to 10% (95% CI: 5%, 16%). Regarding the two excluded studies, one focused on patients with ASS with severe interstitial lung disease (ILD), who had poor basic health conditions and six patients had unexplained fever and increased CRP levels (41). In the other study, 16.7% of patients had a history of cancer, and 30% had a systemic disease. These patients have a high incidence of adverse events because of their poor overall health status (30).

Severe adverse events were defined as events that required hospitalization. The incidence of severe adverse events was determined from seven trials (n = 248). The pooled incidence estimate was 8% (95% CI: 2%, 17%) (**Figure 3B** and **Table 2**). Heterogeneity was significant (*I^2^* = 74.8%, *P* = 0.001). A sensitivity analysis was conducted. After excluding two studies (16, 26), the heterogeneity was resolved (*I^2^* = 0, *P* = 0.715), and the incidence of severe adverse events decreased to 3% (95% CI: 1%, 8%). Regarding the two excluded studies, one was a large randomized, placebo-controlled clinical trial (16), while the other was not a randomized controlled trial (RCT) (26). This difference may have led to the heterogeneity. The latter study (26) focused on patients with ASS and severe ILD, who were prone to severe adverse events, which may have further contributed to heterogeneity.

#### 3.3.2 Incidence of infections and severe infections

Fifteen trials reported the incidence of infections (n = 386). The pooled incidence estimate was 26% (95% CI: 17%, 35%) (**Figure 3C** and **Table 2**). Moderate heterogeneity was detected (*I^2^* = 52.1%, *P* = 0.022). After conducting sensitivity analyses, one study was excluded (41), and the heterogeneity decreased (*I^2^* = 33.3%, *P* = 0.142). The incidence of infections also decreased to 23% (95% CI: 16%, 31%). In the above-mentioned paper, seven patients developed an infection including one patient with *Pneumocystis jirovecii* infection, while the other six patients showed fever and increased CRP without definite infection foci. These differences may have contributed to the heterogeneity.

Severe infection was defined as one that required hospitalization and/or intravenous antibiotic therapy. The incidence of severe infections was determined from 5 trials (n = 81). The pooled incidence estimate was 2% (95% CI: 0, 6%) (**Figure 3D** and **Table 2**). No heterogeneity was observed (*I^2^* = 0, *P* = 0.578).

#### 3.3.3 Incidence of infusion reactions

The incidence of infusion reactions was determined in seven trials (n = 245). The pooled incidence estimate was 12% (95% CI: 6%, 20%) (**Figure 3E** and **Table 2**). No heterogeneity was observed (*I^2^* = 25.4%, *P* = 0.244).

### 3.4 Subgroup analyses

#### 3.4.1 Effectiveness of RTX treatment in patients with different IIM subtypes

**Figure 4:**
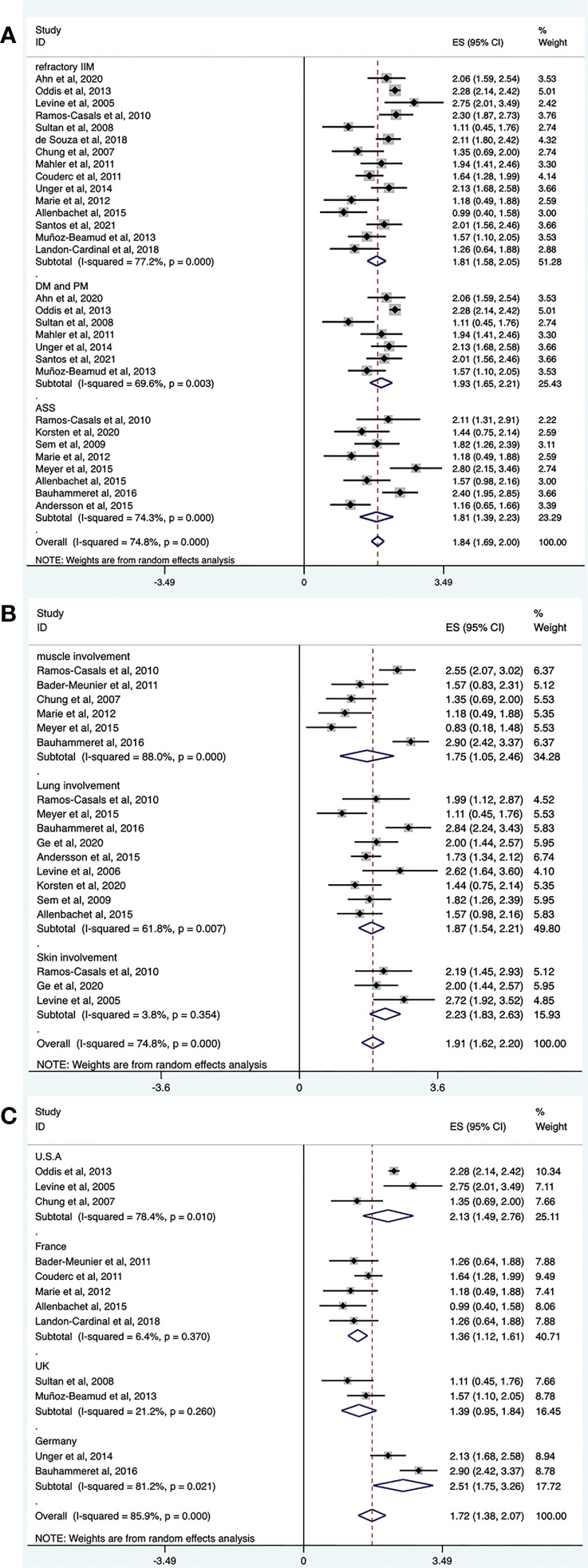
Forest plots of subgroup analysis results. RTX efficacy in patients with different IIM subtypes **(A)**, organ-specific response of RTX in IIMs **(B)**, efficacy of RTX in IIMs from different continents **(C)**.

Refractory IIM was defined as a failure to respond to or tolerate glucocorticoids, combined with at least one of the other standard immunosuppressive or immunomodulatory agents (e.g., azathioprine, methotrexate, mycophenolate mofetil, cyclosporine, tacrolimus, or intravenous immunoglobulin [IVIg]). The overall efficacy rate of RTX in patients with refractory IIMs was determined from 15 trials (n = 412). The pooled effectiveness estimate was 62% (95% CI: 50%, 73%) (**Figure 4A** and **Table 2**). Significant heterogeneity was detected (*I^2^* = 77.2%, *P* = 0.000). Hence, we conducted a sensitivity analysis but studies that contributed to heterogeneity, were not found.

Among the included studies, several regarded patients with DM and PM together. Therefore, we evaluated the effectiveness of RTX in these populations. The overall efficacy rate was determined from seven trials (n = 284). The pooled effectiveness estimate was 68% (95% CI: 54%, 80%) (**Figure 4A** and **Table 2**). Significant heterogeneity was detected (*I^2^* = 69.6%, *P* = 0.003). After conducting sensitivity analyses, two studies were excluded (39, 43). The heterogeneity was resolved (*I^2^* = 0, *P* = 0.528), and the effective rate increased to 80% (95% CI: 75%, 85%). The mean disease duration of the two excluded articles was longer than that in other studies. One was 9.75 years (39) and the other was 14.9 years (43). This may have contributed to a poor response to RTX treatment and to heterogeneity.

The overall efficacy rate of RTX in ASS was determined from eight trials (n = 80), and the pooled effectiveness estimate was 62% (95% CI: 41%, 81%) (**Figure 4A** and **Table 2**). Significant heterogeneity was detected (*I^2^* = 74.3%, *P* = 0.000). After conducting sensitivity analyses, two studies were excluded (22, 36), and the heterogeneity decreased (*I^2^* = 18.1%, *P* = 0.296). The effective rate became 47% (95% CI: 33%, 61%). Regarding the two excluded studies, one focused on Jo-1 antibody–associated ASS and the other focused on anticitrullinated peptide/protein antibody (ACPA)-associated ASS. The remaining six studies targeted patients with ASS without mentioning specific antibodies. The original data suggested that patients with ASS patients who were Jo-1-positive or ACPA-positive responded well to the treatment effect of RTX. These differences may have contributed to the heterogeneity.

#### 3.4.2 Effectiveness of RTX treatment in patients with IIM: The organ-specific responses

Among the included studies, there were several evaluated organ-specific responses. Muscle strength with manual muscle testing (MMT) and/or CK levels were recorded to assess muscle improvement. Six articles (n = 61) assessed the rate of muscle improvement in patients with myositis after RTX treatment, and the pooled estimate of the muscle improvement rate was 59% (95% CI: 25%, 89%; *I^2^* = 88%, *P* = 0.000). ILD was defined as ground-glass changes and/or fibrosis on high-resolution chest computed tomography (HRCT). Regarding the efficacy of RTX for patients with IIM and ILD, pulmonary function tests and/or radiographic changes on HRCT were performed before and after RTX treatment in the included studies. Nine studies (n = 88) assessed the rate of lung improvement after RTX treatment, and the pooled estimate of the improvement rate was 65% (95% CI: 48%, 80%; *I^2^* = 61.8%, *P* = 0.007). Three studies (n = 22) assessed the rate of skin improvement, and the pooled estimate was 81% (95% CI: 63%–94%; *I^2^* = 3.8%, *P* = 0.354) (**Figure 4B** and **Table 2**).

#### 3.4.3 Effectiveness of RTX treatment in patients with IIM from different continents and countries

The included studies did not provide the ethnicity of the patients. Most of the included studies were conducted in Europe and America. Therefore, we performed a subgroup analysis according to the region where the study was conducted rather than race. Among the included studies, 11 trials were conducted in Europe (n = 156), and the pooled estimate of the overall efficacy rate was 56% (95% CI: 39%, 73%). Of these, five studies were performed in France (n = 65) and the overall effective rate was 40% (95% CI: 28%, 52%). Two studies were done in the UK (n = 24) and the overall effective rate of 41% (95% CI: 21%, 63%). Two other studies were from Germany (n = 34) and the overall effective rate of 90% (95% CI: 59%, 100%). Six trials were from America, and the pooled estimate of the overall effective rate was 77% (95% CI: 67%, 85%). Among these, three studies were performed in the United States (n = 209), and the overall efficacy rate was 77% (95% CI: 46%, 96%) (**Figure 4C** and **Table 2**).

### 3.5 Evaluation for publication bias and meta-regression

**Figure 5:**
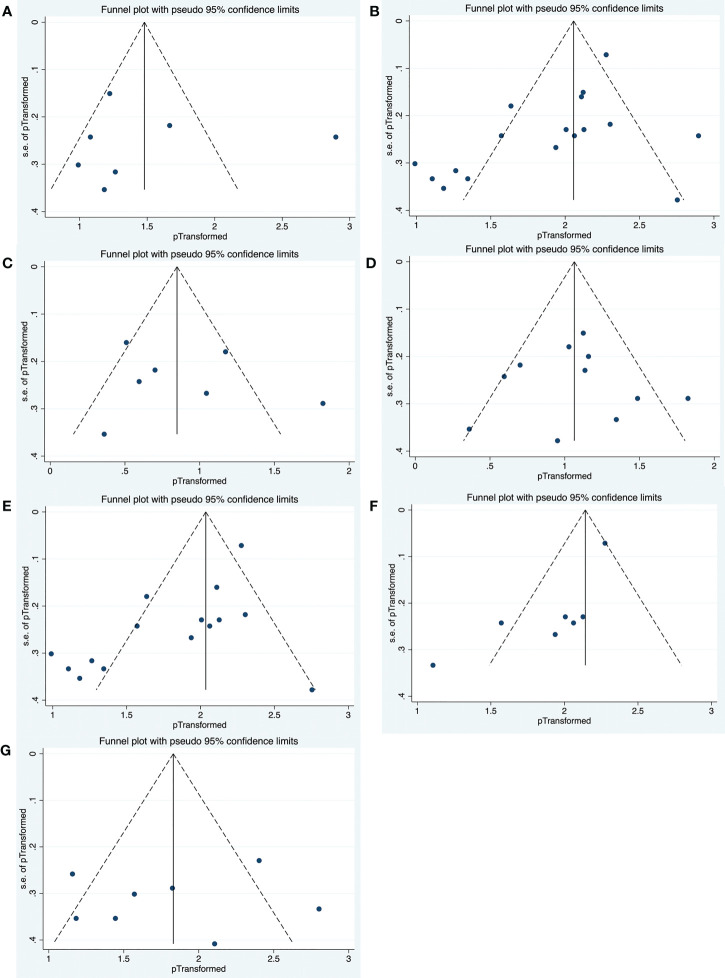
Funnel plots of publication bias in complete response rate group **(A)**, overall effective rate group **(B)**, adverse events group **(C)**, infection group **(D)**, refractory IIM group **(E)**, DM and PM group **(F)**, as well as ASS group **(G)**.

Funnel plot analyses and Begg’s test for publication bias were performed, and the results are presented in **Figure 5**. Egger’s test showed no apparent publication bias in the complete response rate group (*p* = 0.930, **Figure 5A**), adverse event group (*p* = 0.627, **Figure 5C**), infection group (*p* = 0.973, **Figure 5D**), and the ASS group (*p* = 0.770, **Figure 5G**). Conversely, there was a publication bias in the overall effective rate group (*p* = 0.013, **Figure 5B**), refractory IIM group (*p* = 0.006, **Figure 5E**), and DM and PM groups (*p* = 0.021, **Figure 5F**). To explore potential sources of heterogeneity, random-effects meta-regression analyses were conducted for the three groups with bias. The independent variables included sample size, study quality, and publication year. None of these variables significantly contributed to the heterogeneity in the overall effective rate group and refractory IIM group (*p* > 0.05). The sample size may be responsible for the heterogeneity in the DM and PM groups (*p* = 0.027).

## 4 Discussion

IIMs are heterogeneous autoimmune disorders with multiple subtypes, including DM, PM, sIBM, IMNM, and ASS. The condition’s low incidence makes it difficult to perform large-scale RCTs. Currently, glucocorticoids are the first-line treatment (45), and classic immunosuppressants, such as methotrexate or azathioprine, are usually used in combination with glucocorticoids as the initial therapy (1). Mycophenolate is a second-line treatment, but for patients with moderate-to-severe myositis associated with ILD, it may be used as the first-line treatment (46, 47). Cyclosporine and tacrolimus are used as second-line treatments for refractory myositis (48, 49). Previously, IVIg was used as a second- or third-line treatment (1). However, based on the positive results reported by the recent ProDERM (Progress in DERMatomyositis) study, the FDA, and European Medicines Agency have approved IVIg administration in adult DM (1). The ProDERM study was a double-blind, randomized, placebo-controlled, multicenter phase III study that assessed the efficacy, safety, and tolerability of IVIg in the management of DM; the percentage of patients who achieved at least minimal improvement at week 16 was significantly greater in the IVIg group than in the placebo group (50). IVIg is now increasingly used as a first-line treatment for IMNM (51, 52). In a cohort study, half of the patients with IMNM used IVIg as a first-line therapy and 92% at the end of the follow-up period for anti-SRP patients. Patients with anti-SRP receiving IVIg showed an obviously higher remission rate than those without IVIg (52). Among the biological agents used as third-line therapies, RTX is the most common. Evidence supporting the current treatment options come from retrospective cohort studies. Thus, robust clinical evidence is lacking and the management of IIM remains challenging.

The pathogenesis of IIM is still unclear. B cells are involved in many autoimmune responses, including the production of autoantibodies, cytokines secretion, antigen presentation, and modulation of T cells functions. Currently, there is increasing evidence suggesting that B lymphocytes may play an important role in the pathogenesis of IIM, which is summarized as follows.

First, B cells are observed in the perivascular infiltrates of the DM muscle tissues (53). BAFF is important for B cell maturation and survival, and is thought to be involved in the production of autoantibodies as well as the activation and differentiation of T cells. Krystufkova et al. showed that the serum BAFF level was significantly higher in patients with IIMs than in healthy individuals (14). The high serum BAFF level was especially demonstrated in patients with DM, anti-Jo-1 autoantibodies, and ILD (14). Moreover, the level of BAFF expression was significantly increased in DM muscles. BAFF was also expressed in the perifascicular muscle fibers, but not in the blood vessels (15). In addition, a study suggested that the BAFF/BAFF-receptor pathway is involved in T and B cell responses in DM (15).

Second, a previous study identified CD138+ plasma cells in IBM and PM muscles. These plasma cells are terminally differentiated B cells (54). Furthermore, several studies have reported that 60%-80% of patients with IIM are positive for autoantibodies (55, 56), including myositis-specific antibodies (MSAs) and myositis-associated autoantibodies (MAAs). Up to 40% of patients with PM and DM test positive for MSAs (57). MSAs are critical for the diagnosis of IIM and correlate with a unique clinicopathological phenotype. The frequent presence of MSAs and MAAs in IIM suggests that B cells play a role in their pathogenesis. The above evidence supports the feasibility of depleting B-cell using RTX as therapy for patients with IIMs.

Treatment efficacy was the main focus of our meta-analysis. In our study, the overall effective rate of rituximab in IIM was 65%, with higher effective rates of 90% and 77% in studies conducted in Germany and the U.S., respectively. The complete response rate of RTX in patients with IIM was 45% and the partial response rate was 39%. Most patients received RTX because of refractory disease or ILD. This meta-analysis demonstrated that RTX was effective in 62% of patients with refractory IIMs and 68% of patients with DM and PM. However, the lack of head-to-head studies and the presence of heterogeneity make it difficult to draw definitive conclusions.

ASS, a more common subtype of IIM, is characterized by myositis, ILD, fever, Raynaud phenomenon, arthritis, and/or mechanic hands. ILD is the most frequent manifestation of the disease. Arthritis, myositis, and ILD are the classical clinical triads of ASS. In our meta-analysis, the overall efficacy rate of RTX in patients with ASS was 62% (95% CI: 41%, 81%).

IIMs are multi-systemic inflammatory disorders involving not only the muscles but also other organs, such as the skin, lungs, heart, joints, and the gastrointestinal tract. Symmetric proximal muscle weakness and myalgia were the most common symptoms. Skin rashes, ILD, and arthritis were also common and may even be the predominant manifestations. Subgroup analysis showed that the organ-specific response to RTX in the muscle was 59%. Moreover, 65% of the patients with ILD responded to treatment. Furthermore, the therapeutic response was excellent for patients with skin involvement (81%).

How to identify which populations with IIM would be the most likely to benefit from receiving treatment with RTX? Are there any clinical and laboratory factors that predict clinical prognosis? First, the presence of MSAs or MAAs may be associated with the clinical response. In Nalotto’s study, five out of six refractory IIM patients achieved significant clinical improvement 6 months after RTX. All five patients were positive for MSAs or MAAs, whereas those without improvement were negative for autoantibodies (58). Aggarwal et al. conducted a *post hoc* analysis of the RIM trial, revealing that the presence of anti-synthetase autoantibodies (predominantly anti-Jo-1) and anti-Mi-2 autoantibodies strongly predicted clinical improvement in refractory myositis patients, whereas the absence of myositis autoantibodies was associated with a worse outcome (59). In a Korean trial, all six ANA-positive patients responded to RTX (four achieved complete response, two achieved partial response), whereas among the four ANA-negative patients, two achieved partial response and the remainder had no response to RTX. All three anti-Jo-1 antibody positive patients achieved a complete response (25). As mentioned above, the frequent presence of MSAs and MAAs in IIM suggests an important role of B cells in the disease; RTX could effectively deplete B cells to make these populations to achieve a better outcome. Second, the JDM subset may have a better response to RTX, which was not attributable to a shorter disease duration, or these populations may have had lower myositis-related damage (59). Third, lower disease damage predicts better outcomes in patients with refractory myositis (59). Patients with dysphagia, a known serious problem in patients with IIM, showed worse outcomes than those treated with RTX (23).

In Nalotto’s study (58), although five patients with positive autoantibodies responded well to RTX, only one exhibited decreased antibody levels after B cell depletion, suggesting that autoantibody levels may not correlate with clinical response. However, Aggarwal et al. showed that the autoantibody levels correlated with the clinical response to RTX in the RIM trial, demonstrating that the four autoantibodies (anti-Jo-1, -SRP, -TIF1-γ, -Mi-2) levels decreased after B cell depletion; the first three correlated with changes in disease activity (60). These contradictory results require verification in additional RCTs.

The safety of rituximab in patients with IIM was another essential component of our meta-analysis and must be considered in clinical practice. We found that the incidence of adverse events was 18% (95% CI: 7%, 33%), and the incidence of infection was 26% (95% CI: 17%, 35%). In addition, the incidences of severe adverse events and infections, were 8% and 2%, respectively. The infusion reaction was thought to be a joint adverse event of RTX. Our meta-analysis showed that the incidence of infusion reactions was 12%. In brief, RTX was considered relatively safe and well-tolerated in patients with IIM.

This meta-analysis had several notable strengths. To the best of our knowledge, this is the first systematic meta-analysis to evaluate the effectiveness and safety of RTX in patients with IIM. Second, we evaluated the overall efficacy rate, complete response rate, partial response rate, and organ-specific response rate to fully assess the efficacy of RTX. Third, we conducted a comprehensive literature search across five databases using a standardized methodology. Finally, strict inclusion and exclusion criteria were used and case reports and case series with fewer than five participants were excluded, to ensure the quality of the included studies.

Our meta-analysis also has several limitations. First, the included studies failed to provide the mean ± standard deviation (SD) of corticosteroid dosage and CK levels before and after RTX treatment. Therefore, the effects of RTX on corticosteroid tapering and the influence of CK levels could not be evaluated. Second, the RTX regime varied among the included studies, which contributed to the heterogeneity. Third, the included studies had inconsistent definitions of complete and partial responses. This may have been another source of heterogeneity. Fourth, most of the included studies were single-arm tests without control groups, and no RCT data could be summarized, which is difficult to circumvent owing to the rarity of IIMs. Therefore, the conclusions of this study should be verified in clinical practice.

## 5 Conclusion

In summary, this meta-analysis suggests that RTX is a feasible treatment option for patients with IIMs. It is effective and relatively safe in this patient population. Future RCTs are required to further evaluate the efficacy and safety of RTX treatment for patients with IIMs.

## Data availability statement

The original contributions presented in the study are included in the article/**Supplementary Material**. Further inquiries can be directed to the corresponding authors.

## Author contributions

CZ had the conception, collected and analyzed the data, and wrote the manuscript. YH collected and analyzed the data. BZ revised the manuscript. XM analyzed the data. CZ and TD had the conception, help the methods and revised the manuscript. All authors contributed to the article and approved the submitted version.
