# Supplementary material for: Efficacy and safety of rituximab treatment in patients with idiopathic inflammatory myopathies: A systematic review and meta-analysis
Source: Front Immunol. 2022 Dec 12;13:1051609. doi: 10.3389/fimmu.2022.1051609 (PMC9791086; doi:10.3389/fimmu.2022.1051609)
Supplement: Supplementary file 1 [file Presentation_1.pdf]

Search strategies for every database are as follows:

Pubmed (from commencement to 21 Jun 2021)

#1. "Myositis"[Mesh]

#2. (((((Inflammatory Myopathies[Title/Abstract]) OR (Idiopathic Inflammatory Myopathies[Title/Abstract])) OR (Dermatomyositis[Title/Abstract])) OR (Polymyositis[Title/Abstract])) OR (immunemediated necrotizing myopathy[Title/Abstract])) #3. ("Myositis"[Mesh]) OR ((((((Inflammatory Myopathies[Title/Abstract]) OR (Idiopathic Inflammatory Myopathies[Title/Abstract])) OR (Dermatomyositis[Title/Abstract])) OR (Polymyositis[Title/Abstract])) OR (immunemediated necrotizing myopathy[Title/Abstract]))

#4. "Rituximab"[Mesh]

#5. ((Mabthera[Title/Abstract]) OR (anti-CD20[Title/Abstract])) OR (Rituxan[Title/Abstract])

#6. ("Rituximab"[Mesh]) OR (((Mabthera[Title/Abstract]) OR (anti-CD20[Title/Abstract])) OR (Rituxan[Title/Abstract]))

#7. (("Myositis"[Mesh]) OR ((((((Inflammatory Myopathies[Title/Abstract]) OR (Idiopathic Inflammatory Myopathies[Title/Abstract])) OR (Dermatomyositis[Title/Abstract])) OR (Polymyositis[Title/Abstract])) OR (immunemediated necrotizing myopathy[Title/Abstract])) AND (("Rituximab"[Mesh]) OR (((Mabthera[Title/Abstract]) OR (anti-CD20[Title/Abstract])) OR (Rituxan[Title/Abstract])))

Medline (from commencement to 21 Jun 2021)

#1. myositis:ab,ti OR 'idiopathic inflammatory myopathies':ab,ti OR 'inflammatory myopathies':ab,ti OR dermatomyositis:ab,ti OR polymyositis:ab,ti OR 'immunemediated necrotizing myopathy':ab,ti

#2. rituximab:ab,ti OR mabthera:ab,ti OR 'anti cd20':ab,ti OR rituxan:ab,ti

#3. #1 AND #2

Cochrane (from commencement to 21 Jun 2021)

#1. MeSH descriptor: [Myositis] explode all trees

#2. (Idiopathic Inflammatory Myopathies):ti,ab,kw OR (Inflammatory Myopathies):ti,ab,kw OR (Dermatomyositis):ti,ab,kw OR (Polymyositis):ti,ab,kw OR (Immunemediated Necrotizing Myopathy)

#3. #1 or #2

#4. MeSH descriptor: [Rituximab] explode all trees

#5. (Mabthera):ti,ab,kw OR (anti-CD20):ti,ab,kw OR (Rituxan):ti,ab,kw

#6. #4 or #5

#3. #3 and #6

WANFANG (from commencement to 21 Jun 2021)

#1. ((((((肌炎) OR 特发性炎性肌病) OR 炎性肌病) OR 皮肌炎) OR 多发性肌炎)) OR 免疫介导坏死性肌炎)))

#2. ((((利妥昔单抗) OR 美罗华) OR 抗 CD20))

#3. #1 AND #2

CNKI (from commencement to 21 Jun 2021)

（((主题=肌炎 或者 题名=肌炎))或者 （主题=特发性炎性肌病 或者 题名=特发性炎性肌病））或者 （主题=炎性肌病 或者 题名=炎性肌病））或者 （主题=皮肌炎 或者 题名=皮肌炎））或者 （主题=多发性肌炎 或者 题名=多发性肌炎））或者 （主题=免疫介导坏死性肌炎 或者 题名=免疫介导坏死性肌炎）)）并且 （((主题=利妥昔单抗 或者 题名=利妥昔单抗）)或者（主题=美罗华 或者 题名=美罗华）)或者（主题=抗 CD20 或者 题名=抗 CD20))）
